# Gambogic Acid Inhibits Wnt/β-catenin Signaling and Induces ER Stress-Mediated Apoptosis in Human Cholangiocarcinoma

**DOI:** 10.31557/APJCP.2021.22.6.1913

**Published:** 2021-06

**Authors:** Kanoknetr Suksen, Keatdamrong Janpipatkul, Somrudee Reabroi, Natthinee Anantachoke, Vichai Reutrakul, Arthit Chairoungdua, Natthakan Thongon, Kanit Bhukhai

**Affiliations:** 1 *Department of Physiology, Faculty of Science, Mahidol University, Bangkok, Thailand. *; 2 *Department of Basic Medical Science, Faculty of Medicine Vajira Hospital, Navamindradhiraj University, Bangkok, Thailand. *; 3 *Department of Pharmacology, Faculty of Science, Mahidol University, Bangkok, Thailand. *; 4 *Department of Pharmacognosy, Faculty of Pharmacy, Mahidol University, Bangkok, Thailand. *; 5 *Department of Chemistry and Center of Excellence for Innovation in Chemistry (PERCH-CIC), Faculty of Science, Mahidol University, Bangkok, Thailand. *; 6 *Excellent Center for Drug Discovery (ECDD), Mahidol University, Bangkok, Thailand. *; 7 *Toxicology Graduate Program, Faculty of Science, Mahidol University, Bangkok, Thailand. *; 8 *Center of Excellence on Environmental Health and Toxicology (EHT), Faculty of Science, Mahidol University, Bangkok, Thailand. *

**Keywords:** Gambogic acid, cholangiocarcinoma, anti-cancer, Wnt/β-catenin signaling pathway, ER stress

## Abstract

**Objective:**

Gambogic acid (GA) has been reported to induce apoptosis in cholangiocarcinoma (CCA) cell lines. However, the molecular mechanisms underlying its anti-cancer activity remain poorly understood. This study was aimed to investigate GA’s effect on human CCA cell lines, KKU-M213 and HuCCA-1, and its associated mechanisms on Wnt/β-catenin signaling pathway.

**Methods:**

Cell viability, apoptosis, and cell cycle analysis were conducted by MTT and flow cytometry. The effect of GA mediated Wnt/β-catenin and ER stress were determined by luciferase-reporter assay, qRT-PCR, and western blot analysis.

**Results:**

GA exhibited potent cytotoxicity in CCA cells which was associated with significantly inhibited cell proliferation, promoted G1 arrest, and activated caspase 3 mediated-apoptosis. GA attenuated β-catenin transcriptional levels, decreased β-catenin protein, and suppressed the expression of *c-Myc*, a downstream target gene of Wnt/β-catenin signaling. GA activated genes involved in ER stress mechanism in KKU-M213 and enhanced CCA’s sensitivity to gemcitabine.

**Conclusion:**

Our findings reveal that the molecular mechanism underpinning anti-cancer effect of GA is partially mediated through the inhibition of Wnt/β-catenin signaling pathway and induction of ER stress induced-apoptosis. GA may serve as a promising therapeutic modality for amelioration of gemcitabine-induced toxicity in CCA.

## Introduction

Cholangiocarcinoma (CCA) is the second most common primary liver tumor with a morphological feature of the aggressive malignant proliferation of biliary duct epithelial cells. While CCA incidence rates are increasing globally, the highest incidence and mortality rates are in the North-eastern Thailand specifically in the endemic area of liver fluke infestations (Banales et al., 2016; Prakobwong et al., 2017; Alsaleh et al., 2019; Chansitthichok et al., 2020). Indeed, surgery is the principal treatment that provided the best survival prognosis of CCA patients (Chanchai et al., 2019), however, 40-85% of all patients have recurrent disease even after tumor excision (Vogel et al., 2014; Marin et al., 2018) and surgical resection has become an incurable option for most CCA patients in advanced stages of the disease (Yao et al., 2014). Due to rapid progression of CCA, therapeutic effort has been directed to identifying and modulating key molecular pathways relevant to cancer growth. Chemotherapy for advanced CCA is largely ineffective due to the emerging of resistance in CCA (Sawasdee et al., 2020). Therefore, targeting essential signaling pathways mediated CCA progression is critical for understanding and developing treatments. 

Wnt/β-catenin signaling pathway plays important roles in normal tissue development, including cell proliferation and differentiation (Nusse and Clevers, 2017). Cytosolic β-catenin protein is a principal mediator of Wnt/β-catenin signaling and its phosphorylation, ubiquitination, and degradation are governed by a destruction complex comprising of Axin, adenomatous polyposis coli (APC), and glycogen synthase kinase-3β (GSK-3β). Aberrant activation of this complex gives rise to the accumulation of β-catenin in nucleus which functions as a transcriptional coactivator of transcription factors included in the T-cell factor and lymphoid enhancer factor (TCF/LEF) family and promotes the transcription of Wnt responsive genes including *c-Myc* and *CCND1*. Hyper-activation of Wnt/β-catenin signaling observe in several types of human cancers which drives tumor initiation and progression (Tripathy et al., 2018). Thus, the discovery of compounds targeting Wnt/β-catenin signaling has potential as a therapeutic strategy for CCA. 

**Figure 1:**
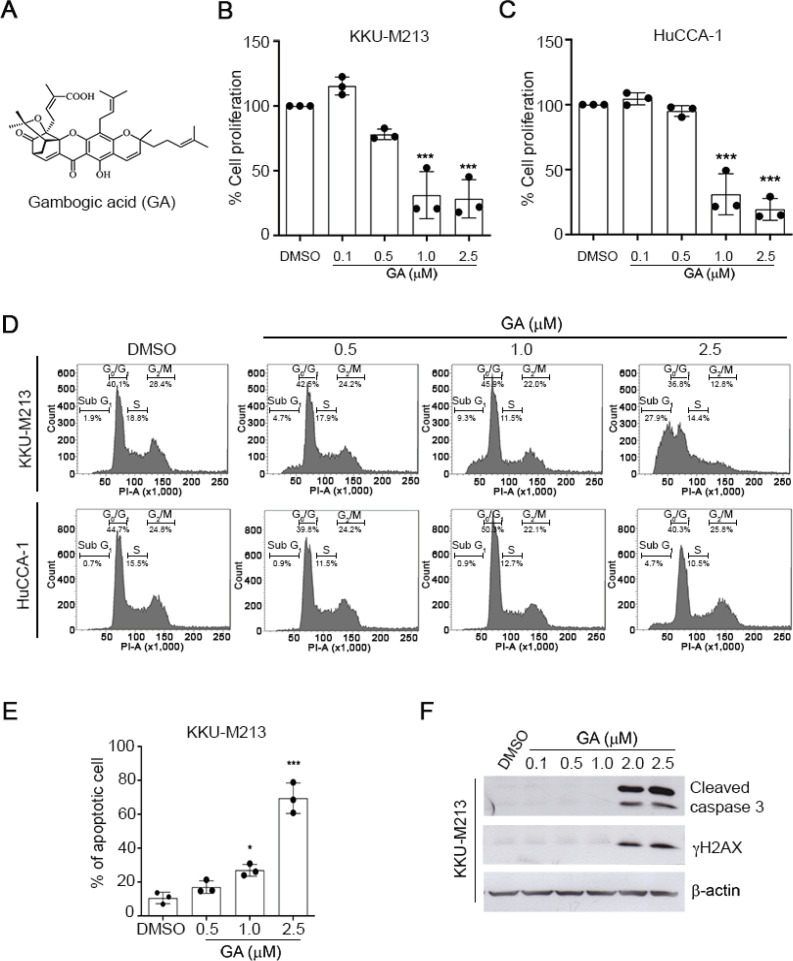
GA Inhibits Cell Proliferation and Induces Apoptosis in CCA. (A) Chemical structures of Gambogic acid (GA) (B-C) The anti-proliferative effect of GA in KKU-M213 and HuCCA-1 cells evaluated by BrdU cell proliferation assay 24 h after treatment. (D) Cell cycle progression of CCA cells treated with GA for 24 h. (E) The percentage of apoptotic cells in GA treated-KKU-M213 at 24 h. (F) Representative Western blot of cleaved-caspase 3 and γH2AX of GA treated-KKU-M213 for 24 h is shown. β-actin was used as a loading control

Xanthone is isolated from the resin of *Garcinia hanburyi *(Reutrakul et al., 2007; Hahnvajanawong et al., 2010). Among the naturally occurring caged xanthones, Gambogic acid (GA) has been identified as an effective anti-cancer agent in a variety of tumors (Zhang et al., 2004; Kashyap et al., 2016). GA has a unique structure in which the planar structure of xanthone (Figure 1A) is recognized as an efficient DNA intercalator possessing anti-cancer activities (Qin et al., 2007) and an intact ABC ring of caged xanthone compounds reveals an optimisation of a given bioactivity against cancers. In particular, the 9, 10 carbon-carbon double bond of the α, β-unsaturated ketone is essential for their cytotoxic and apoptotic activities (Zhang et al., 2004). Accumulating evidence indicates that GA strongly induces ER stress, suppresses human growth factor receptors (He et al., 2013), and regulates several signaling pathways which are crucial for its molecular processes in cancers. However, the specific molecular mechanisms related to anti-cancer activity of GA in CCA cells are still unclear. Here we aimed to investigate the effect of GA on the modulation of Wnt/β-catenin signaling pathway in CCA malignancy phenotypes. 

## Materials and Methods

*Chemicals, plasmids, and reagents*

Gambogic acid (GA) (Figure 1A) was isolated from Garcinia hanburyi (Family Guttiferae) as previously described (Reutrakul et al., 2007). Dual-luciferase reporter assay was purchased from Promega (Madison, WI). β-catenin-pcDNA3.1(+), TOPflash, FOPflash, S33Y, and Renilla were previously described elsewhere (Chairoungdua et al., 2010). The following reagents were used: MTT (3-(4, 5-dimethylthiazol-2-yl)-2, 5-diphenyl tetrasolium bromide) (Sigma-Aldrich, St. Louis, MO) and cell Proliferation ELISA and BrdU colorimetric assay were from Roche (Roche, Mannheim, Germany).

*Cell culture*

KKU-M213 cells were purchased from JCRB (Osaka, Japan). HEK 293T cells were obtained from American Type Culture Collection (ATCC, Manassas, VA). HuCCA-1 cell line was kindly provided by Dr. Stitaya Sirisinha, Department of Microbiology, Faculty of Science, Mahidol University. KKU-M213 and HuCCA-1 cells were cultured in Ham’s F-12 medium (Invitrogen, Carlsbad, CA). HEK 293T cells were maintained in Minimum Essential Medium (MEM). All culture mediums were supplemented with 10% Fetal bovine serum (FBS) and 100 U/ml penicillin/streptomycin and cells were cultured at 37°C in a humidified atmosphere containing 5% CO_2_ incubator. 

*Cell proliferation and cytotoxic assay*

Cell proliferation was determined by a Cell Proliferation ELISA and BrdU colorimetric assay according to the manufacturer’s instructions. GA treated-cells were incubated in BrdU labelling solution for 2 h at 37°C and BrdU-POD antibody was added and incubated for 90 min at room temperature (RT). Cell proliferation was determined by measuring the absorbance at 450 nm using a Microplate Spectrophotometer. Cell viability was determined by MTT assay. Briefly, cells were incubated with 0.5% MTT reagent for 4 h at 37°C to allow the formation of a violet formazan precipitate. Then, supernatant was removed and formazan crystals were dissolved in 100% DMSO prior to measuring absorbance at 540 nm by a Multiskan GO Microplate Spectrophotometer (Thermo Scientific, Waltham, MA). 

*Annexin V and Propidium iodide (PI) staining*

Cells were treated with GA for 24 h. They were harvested and incubated with Annexin V-FITC and PI for 15 min at RT in the dark. The frequency of apoptotic cells was determined by BD FACSCanto™ flow cytometer and analyzed with BD FACSDiva software (BD Bioscience, San Jose, CA).

*Cell cycle analysis*

CCA cells were treated with GA for 24 h, fixed with cold 70% ethanol overnight at 20°C and stained in PI/RNase Staining Buffer in the dark for 30 min at 37°C. Cell cycle analysis was performed by BD FACSCanto™ flow cytometer and analysed with a BD FACSDiva software.

*Luciferase reporter assay*

HEK 293T cells were transiently transfected with FLAG-β-catenin, TOPflash/TCF reporter, FOPflash, and Renilla luciferase reporter plasmids using lipofectamine 3000 according to the manufacturer’s instructions (Invitrogen, Carlsbad, CA). Twenty-four hours after transfection, cells were treated with GA for 24 h and subjected to reporter assay analysis using Dual-luciferase reporter kit.

*Western blot analysis*

Total protein lysates were harvested using a modified lysis buffer as previously described (Bhukhai et al., 2012). Samples were resolved by 10% of sodium dodecyl sulfate polyacrylamide gel electrophoresis (SDS-PAGE). The following antibodies were used: β-catenin (H-102) (Santa Cruz Biotechnology, CA), β-actin (Sigma-Aldrich), Cleaved-caspase 3, p-GSK3 (Ser 9), and gH2AX (Cell signaling, MA). Membranes were then probed with HRP-conjugated secondary antibodies for 1 h. The signal was detected using SuperSignal West Pico Chemiluminescent ECL system (Thermo Scientific, Waltham, MA).

*RNA extraction and real time PCR*

Total RNAs were isolated using Trizol reagent according to the manufacturer’s instructions. The quantity of total RNA was determined by NanoDrop 2000 (Thermo Scientific, Waltham, MA). First-strand complementary DNA (cDNA) synthesis was performed using iScriptTM cDNA synthesis kit (Bio-Rad, Hercules, CA). For real-time quantitative PCR, cDNA samples were amplified using SYBR Green I dye and conducted with ABI PRISM7500 Sequence Detection System and analysis software (Applied Biosystem; Bedford, MA). The mRNA expression was normalized to the levels of endogenous *GAPDH* and sequences of primers are *GRP78* (FW: CCCGAGAACACGGTCTTTGA, RW: TCAACCACCTTGAACGGCAA), *IRE-1* (FW: CGGCCTCGGGATTTTTGGAA, RW: TTGAGCCTGTCCTCTTGCTG), *XBP-1* (FW: GGAAGCCAAGGGGAATGAAGT, RW: GCTGCAGAGGTGCACGTAG), *CHOP* (FW: GGAACCTGAGGAGAGAGTGTTC, RW: TGCCATCTCTGCAGTTGGAT), and *GAPDH* (FW: ATGCCCCCATGTTCGTCATG, RW: GCAGGAGGCATTGCTGAT).

*Statistical analysis*

Data are represented as mean and standard deviation (mean ± SD) or mean ± standard error of mean (SEM) for IC50 (Supplementary Table 1). Data were analysed using the statistical software package, GraphPad Prism version 8.2 (GraphPad, CA, USA). The statistical differences among groups were analysed by a one-way ANOVA followed by Tukey-Kramer post-hoc test and paired t-test was used when applicable. Each value is represented as means ± SD of three independent experiments. Statistical significance was considered when values of *, p < 0.05, **, p < 0.01, ***, p < 0.001 compared with vehicle control. 

## Results

*GA exhibits cytotoxic and anti-proliferative effects in CCA cells*

To investigate the effects of GA in CCA, KKU-M213 and HuCCA-1 cells were treated with various concentrations of GA for 24-72 h and cell viability was determined by MTT assay. Exposure of GA markedly reduced cell viability in time and dose-dependent manners in both CCA cell lines. The IC50 values of GA in KKU-M213 and HuCCA-1 cells are shown in Supplementary Table 1. These results aligned with a significantly decreased cell proliferation assessed by a BrdU assay (Figures 1B and 1C). GA significantly inhibited KKU-M213 and HuCCA-1 cell proliferation (p < 0.01) suggesting the potential role of GA in inhibiting cell viability and proliferation of CCAs. 

*GA induces cell cycle arrest and apoptosis in CCA cells*

To demonstrate the effect of GA on cell cycle distribution, GA-treated CCA cells were subjected to flow cytometry analysis. As shown in Figure 1D, GA increased the accumulation of KKU-M213 cells at sub G1 phase in a dose-dependent manner. The frequency of sub G1 cells was increased from 1.9% in the vehicle control to 4.7%, 9.3%, and 27.9% by treatments of GA at 0.5, 1.0, and 2.5 µM, respectively. In contrast, the effect of GA on cell cycle arrest was less significant in HuCCA-1 cells than KKU-M213. We further investigated whether GA induces apoptotic cell death in CCA cells. Flow cytometry analysis of GA-treated CCA cells doubly labelled with annexin V and PI revealed that the frequency of annexin V positive cells was significantly increased in KKU-M213 cells by GA (Figure 1E). Despite GA exhibited cytotoxicity and anti-proliferative effects in HuCCA-1 cells, but GA (at 0.5-2.5 µM) failed to induce apoptosis and cell cycle arrest in HuCCA-1 cells (Figure S1A) suggesting that the sensitivity to GA might be due to a cell line-dependence. In addition, we found that GA induced the activation of gH2AX, a biomarker of DNA double-strand breaks (Figure 1F). This finding accompanied with an increase in the number of cells with nuclear fragmentation and chromatin condensation, a hallmark of apoptosis GA-treated CCA cells (Figure S1B) indicating the potential role of GA inducing DNA damage-mediated cell death. Consistently, GA-induced apoptosis was confirmed by increased cleaved-caspase 3 in KKU-M213 cells (Figure 1F). These results strongly suggest that GA induces DNA damage activation and possesses its anti-cancer effect through the induction of caspase 3-dependent apoptosis in KKU-M213 cells. 

*GA inhibits Wnt/β-catenin signaling*

**Figure 2:**
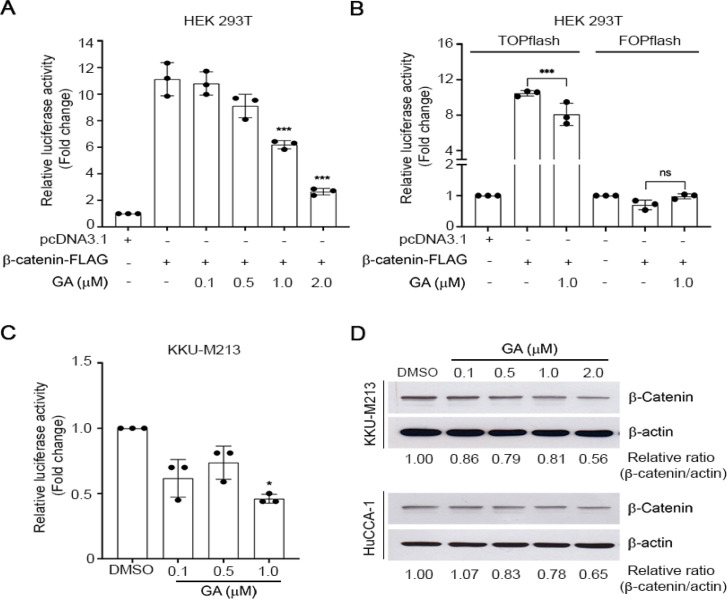
GA Attenuates TCF/LEF Reporter Activity and β-catenin Protein Expression. TCF/LEF reporter activity of GA treated HEK 293T cells over expressing TOPflash (A), TOPflash or FOPflash (B) for 24h. (C) TCF/LEF luciferase activity of GA treated KKU-M213 cells. Relative luciferase activity was quantified by measuring the relative firefly luciferase activity units and normalized to Renilla luciferase activity. (D) Representative Western blot of β-catenin in GA treated KKU-M213 and HuCCA-1 for 24 h

Wnt/β-catenin signaling pathway is activated in multiple cancers (Tripathy et al., 2018) and caspase 3-dependent apoptosis has been reported to be partially mediated through Wnt/β-catenin signaling pathway (Wu et al., 2014). To address the probable anti-cancer mechanism of GA on Wnt/β-catenin signaling pathway, we examined the effect of GA mediated-Wnt/β-catenin transcriptional activity using a luciferase reporter assay in HEK 293T cells which is lack of endogenous levels of β-catenin. Cells were transiently transfected with TOPflash, FLAG-β-catenin, and Renilla plasmids, and then treated with GA. We observed that TCF/β-catenin transcriptional activity was significantly inhibited by GA in a dose-dependent manner (Figure 2A). The inhibitory effect of GA on β-catenin dependent signaling was further confirmed by using FOPflash, an LEF mutant reporter plasmid, by which the expression of β-catenin failed to induce Wnt/β-catenin transcriptional activity. As expected, GA did not inhibit Wnt/β-catenin activity in FOPflash transfected HEK 293T cells (Figure 2B) indicating the modulation of GA on this pathway is β-catenin dependent-TCF activation. We next performed the TCF/LEF-luciferase reporter assay in KKU-M213 and HuCCA-1 cells. A sublethal concentration of GA at 1 µM was administrated to the cells and we observed that GA at 1 µM significantly attenuated reporter activity in KKU-M213 (Figure 2C) as well as the trend of reduction of reporter activity was noticed in HuCCA-1 cells (Figure S1C). We next examined the level of β-catenin protein, which is a key component in Wnt/β-catenin signaling pathway. GA slightly attenuated β-catenin protein levels in KKU-M213 (Figure 2D). These results suggested the inhibitory effect of GA on Wnt/β-catenin signaling in KKU-M213.

β-catenin phosphorylation is mediated by GSK-3β and it results in targeting for degradation by the proteasome pathway (Nusse and Clevers, 2017). Mechanistically, to address whether GA attenuated-Wnt/β-catenin signaling is mediated through GSK-3β, we evaluated the effect of GA on Wnt/β-catenin signaling using a constitutively active β-catenin mutant, S33Y in HEK 293T cells. S33Y is insensitive to GSK-3β-mediated phosphorylation, thereby blocking proteasome-induced protein degradation. We observed that the sublethal concentration of GA at 1 µM significantly decreased Wnt/β-catenin activity in HEK 293T cells overexpressing wild-type β-catenin (Figure S2A) but did not affected in S33Y-β-catenin mutant. These results were confirmed by co-treatment with LiCl, a known GSK-3β inhibitor and we observed that LiCl treatment resulted in greater activation of TOPflash luciferase activity. However, GA failed to inhibit Wnt/β-catenin signaling in the presence of LiCl in HEK 293T cells (Figure S2B) suggesting that GA inhibited Wnt/β-catenin signaling is a GSK-3β dependence activation in HEK 293T cells. We further determined the effect of GA on phosphorylation GSK-3β (Ser 9) protein in KKU-M213. In the contrary to HEK 293T, exposure to GA did not alter the levels of p-GSK-3β in KKU-M213 (Figure S2C). This result implies that inhibition of Wnt/β-catenin signaling by GA in KKU-M213 cells might be independent from GSK-3β’s action. We speculated that the modulation of GA on the GSK-3β is a cell line specific. However, the pathophysiological consequences of Wnt/β-catenin signaling mainly depend on the expression of downstream target genes (Wang et al., 2015). To address this significance, we therefore investigated the effect of GA on the expression of downstream Wnt target genes including *AXIN2* and *c-Myc*. GA induced a dose-dependent activation of *AXIN2*, a negative feedback regulator of Wnt/β-catenin signaling (Figure S2D), whereas markedly suppressed *c-Myc* mRNA expression (Figure S2E). These results indicate that GA potentially inhibited the TCF/LEF-dependent activation of the canonical Wnt/β-catenin signaling and its downstream target gene *c-Myc*. 

*GA induces endoplasmic reticulum (ER) stress in CCA cells*

**Figure 3:**
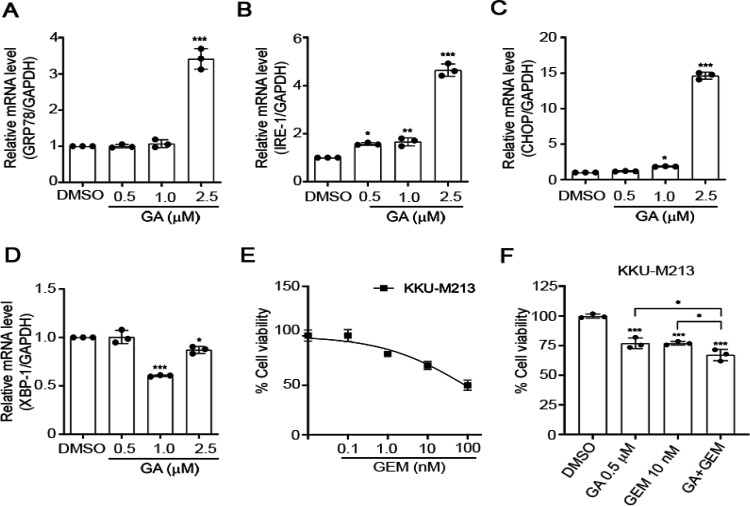
GA Induces Endoplasmic Reticulum (ER) Stress in CCA Cells. KKU-M213 cells were treated with GA for 24 h and quantitative RT-PCR for ER stress target genes, *GRP78/BiP* (A), *IRE1α* (B),* CHOP* (C), and *XBP1* (D) were performed. Data are expressed as fold change compared with those treated with vehicle control and represented as means ± SD (n = 3). *GAPDH* was used as a housekeeping gene. (E) Cell viability of GEM treated KKU-M213 for 72 h. (F) Cell viability experiment of KKU-M213 treated with GA (0.5 μM) alone or in combination with GEM (10 nM) for 72 h. *, p < 0.05 compared to indicated treatments, ***, p < 0.001 compared with vehicle control (ANOVA).

Sustained ER stress results in cancer cell death and impairs Wnt protein processing (Verras et al., 2008). ER stress induced-apoptotic cell death h as been reported in CCA (Vaeteewoottacharn et al., 2013). To determine whether GA induces ER stress promoted-apoptosis cell death in KKU-M 213cells, the mRNA levels of critical genes involved in ER stress mechanism including *GRP78/BiP, IRE1α, XBP1*, and *CHOP* were determined. *GRP78/BiP, IRE1α,* and *CHOP* mRNAs were significantly up-regulated in KKU-M213 cells exposed to GA for 24 h, with a maximal functional response at 2.5 μM (Figure 3A - 3C) in accordance with our previous experiment showing that caspase 3-dependent apoptosis was induced by GA. It should be noted that the *XBP1* mRNA was significantly decreased by 1-2.5 μM of GA treatment (Figure 3D). These results demonstrate that GA induced ER stress-mediated apoptosis in KKU-M213 cells. 

*GA enhances the sensitivity of CCA cells to Gemcitabine*

Gemcitabine (GEM) has been used as an established regimen for advanced biliary cancer (Abdel-Rahman et al., 2018). GEM decreased cell viability of KKU-M213 in a dose-dependent manner (Figure 3E). To evaluate whether GA efficiently enhances CCA cell’s sensitivity to GEM, KKU-M213 cells were treated with GA alone or in combination with GEM for 72 h. We observed that GA sensitized GEM inhibition CCA cell viability (Figure 3F). These results highlight the possibility that the combination treatment of GA and GEM can potentially improve CAA treatment.

**Graph 1:**
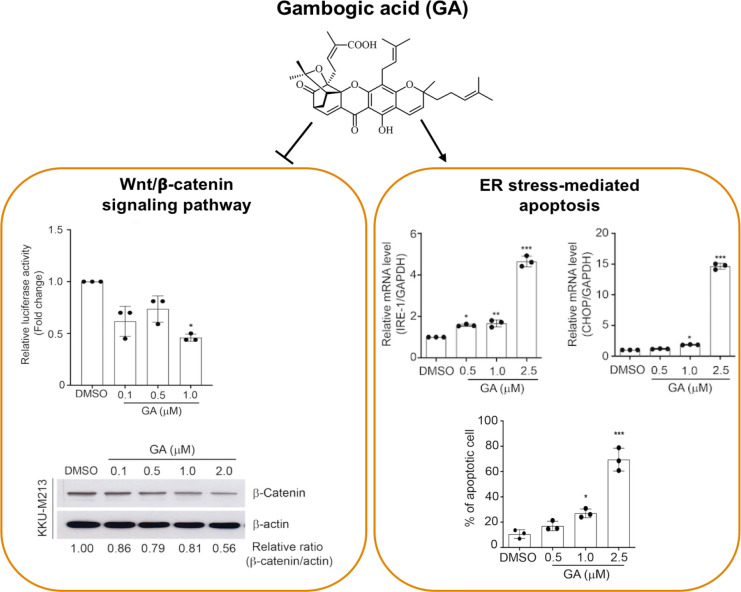
Graphical Abstract

## Discussion

In this present study, we demonstrated that the anti-cancer effects of GA against KKU-M213 partially mediates through the inhibition of Wnt/β-catenin signaling pathway. GA potently inhibited CCA cell proliferation, arrested cell cycle, and substantially induced ER stress and apoptosis which significantly impacted on CCA cell survival. To our knowledge, this is the first report showing that the inhibitory effect of GA on Wnt/β-catenin signaling pathway and its activation to induce ER stress mediated-apoptosis may cooperate to suppress the progression of CCA cells. 

We observed potent cytotoxic effect of GA in both KKU-M213 and HuCCA-1 cell lines. Despite GA exhibited cytotoxicity and anti-proliferative effect in HuCCA-1 cells, but GA (at 0.5 - 2.5 µM) failed to induce apoptosis and cell cycle arrest in HuCCA-1 cells (Figure S1A) which raises an intriguing question of whether acquired resistant mechanism mediated by GA is a cell line dependence. However, further investigation of the molecular mechanisms govern HuCCA-1 resistance should be addressed.

Aberrant activation of Wnt/β-catenin signaling cascade is implicated in a variety of tumors, including hepatocellular tumor (Nejak-Bowen and Monga, 2011) and CCA (Boulter et al., 2015). Unlike in HEK293 cells, we observed that the inhibition of β-catenin was not directly regulated by the phosphorylation of GSK-3β in CCA (Figure S2C). We thus anticipated the possibility that GA-inhibited β-catenin expression in this cell line may be regulated by other components of Wnt signaling pathway such as *AXIN2* which is one of an important Wnt/β-catenin target gene and acts as a negative regulator of Wnt/β-catenin signaling through a negative feedback mechanism (Jho et al., 2002). The expression of *AXIN2 *was up-regulated during activation of Wnt/β-catenin signaling to promote the differentiation of mouse pre-osteoblastic cells (Bhukhai et al., 2012), while in our current study, *AXIN2* was significantly increased, despite a decrease in β-catenin protein expression by GA in KKU-M213 cells. The results corroborate well with the previous study showing that *AXIN2* has been demonstrated to promote degradation of β-catenin (Bernkopf et al., 2015). At present, the underlying mechanism is still unclear. However, elucidating the effect of GA on upstream components of Wnt signaling pathway which directly regulate the degradation of β-catenin should be considered in the future for a better understanding of the molecular mechanisms of GA.

*CHOP* is one of the key downstream molecules involved in ER stress-mediated apoptosis (Han et al., 2019). It should be noted that the *XBP1* mRNA was significantly decreased by GA (Figure 3D) but there was a significantly increased *CHOP* mRNA expression (Figure 3C). These data were consistent with a previous study showing that knockdown of *XBP1* significantly up-regulated *CHOP* protein leading to an increase in apoptosis rate and cell cycle arrests in mouse granulosa cells (Wang et al., 2017). Thus, we anticipated that the reduction of *XBP1* by GA may cooperate the result in the induction of apoptosis and cell cycle arrest through activating* CHOP*. In addition, GEM is a first-line therapy for pancreatic ductal adenocarcinoma and it has been explored as a single agent or in combination treatments (Gulhati et al., 2019). However, gemcitabine resistance appears to be a major obstacle of chemotherapy in CCA (Sawasdee et al., 2020). Here we showed that GA sensitized CCA cells to GEM (Figure 3F) and the combination of GA could be envisaged to overcome gemcitabine resistance in CAA patients.

In summary, our data demonstrates the significant biological activities of GA against CCA cell growth through the inhibition of Wnt/β-catenin signaling and modulation of ER stress induced-apoptosis. GA holds promise as an anticancer and it may represent as a novel therapeutic option for CCA which could be exploited to minimize the use of gemcitabine in the setting of the combination treatment with the current chemotherapy.

## Author Contribution Statement

K.B., K.S., K.J., and A.C. conceived and planned the experiments. K.S, K.J., S.R., and K.B. performed the experiments. K.B., K.S, K.J., N.A., V.R., N.T., and A.C contributed to analysis and interpretation of the results. K.B., K.S, K.J., N.T., and A.C wrote the original manuscript. All authors reviewed and approved the final manuscript
